# The growth factor multimodality on treating human dental mesenchymal stem cells: a systematic review

**DOI:** 10.1186/s12903-024-04013-2

**Published:** 2024-03-01

**Authors:** Huiying He, Yun-Hsuan Yang, Xuesong Yang, Yue Huang

**Affiliations:** 1 School of Stomatology, Jinan University, Guangzhou, 510632 China; 2 Clinical Research Center, Clifford Hospital, Guangzhou, 511495 China

**Keywords:** Dental mesenchymal stem cells, Growth factors, Growth factor receptors

## Abstract

**Background:**

Ensuring the quantity, quality, and efficacy of human dental mesenchymal stem cells (MSCs) has become an urgent problem as their applications increase. Growth factors (GFs) have low toxicity, good biocompatibility, and regulate stem cell survival and differentiation. They bind to specific receptors on target cells, initiating signal transduction and triggering biological functions. So far, relatively few studies have been conducted to summarize the effect of different GFs on the application of dental MSCs. We have reviewed the literature from the past decade to examine the effectiveness and mechanism of applying one or multiple GFs to human dental MSCs. Our review is based on the premise that a single dental MSC cannot fulfill all applications and that different dental MSCs react differently to GFs.

**Methods:**

A search for published articles was carried out using the Web of Science core collection and PubMed. The study was conducted following the Preferred Reporting Items for Systematic Reviews and Meta-Analyses (PRISMA 2020) guidelines. This review considered studies from 2014 to 2023 that examined the effects of GFs on human dental MSCs. The final selection of articles was made on the 15th of July 2023.

**Results:**

Three thousand eight hundred sixty-seven pieces of literature were gathered for this systematic review initially, only 56 of them were selected based on their focus on the effects of GFs during the application of human dental MSCs. Out of the 56, 32 literature pieces were focused on a single growth factor while 24 were focused on multiple growth factors. This study shows that GFs can regulate human dental MSCs through a multi-way processing manner.

**Conclusion:**

Multimodal treatment of GFs can effectively regulate human dental MSCs, ensuring stem cell quality, quantity, and curative effects.

**Supplementary Information:**

The online version contains supplementary material available at 10.1186/s12903-024-04013-2.

## Background

Human dental mesenchymal stem cells (MSCs) are abundant and easily accessible multipotent cells compared to other types of MSCs, such as bone marrow or umbilical cord MSCs. Dental MSCs have significant advantages including the ability to self-renew, regulate the immune system, and differentiate in multiple directions. There are various types of dental stem cells, such as periodontal ligament stem cell (PDLSC), dental follicle stem cell (DFSC), gingival mesenchymal stem cell (GMSC), stem cell from human exfoliated deciduous teeth (SHED), dental pulp stem cell (DPSC), and stem cell of the apical papilla (SCAP) [1]. These cells could be used in cellular therapy, and their development could lead to techniques used in regenerative dentistry and the treatment of degenerative diseases [2, 3].

Various methods have been used to enhance the activity of stem cells, including immortalization, hypoxia, drugs, chemical reagents, physical factors, cytokines, and growth factors (GFs) [4, 5]. GFs have been extensively studied for their good biocompatibility and low toxicity, as they are important regulators of MSCs. The most studied types of GFs are transforming growth factor-β (TGF-β), vascular endothelial growth factor (VEGF), bone morphogenetic protein (BMP), fibroblast growth factor (FGF), platelet-derived growth factor (PDGF), and nerve growth factor (NGF). However, different dental MSCs respond differently to a single GF. For instance, TGF-β or PDGF can stimulate the proliferation and chemotaxis of PDLSC, while they show the effect of chemotaxis on GMSC [6–9]. The combined application of GFs can promote the realization of functions synchronously and sequentially, even if they are not initiated separately [10].

GFs play a crucial role in regulating the biological responses of stem cells. When GFs bind to receptors present on the surface or cytoplasm of target cells, they trigger various signal transduction mechanisms that perform specific functions. For instance, GFs activate odontogenic differentiation through pathways such as ALK5/Smad2/3, TAK1, p38, and MEK/ERK signaling [11, 12]. BMPs also bind to specific type I and type II serine/threonine kinase receptors to activate downstream expression, and it is the type I receptor that determines the nature of the biological response [13]. The activation of specific Smad molecules may vary over time and space, which is why BMP-2/-7 requires precise temporal and spatial regulation to induce the correct biological response [8, 9].

However, one dental MSC source cannot meet all the application requirements. The traditional culture process of dental MSCs has several risks including the transmission of prion and zoonotic viruses due to animal serum. Additionally, there are problems with aging, as well as the low differentiation rate and high apoptosis rate of MSCs [4, 14, 15]. It is necessary to advance the known growth factors and signaling molecules implicated in tooth development and regeneration of different structures of teeth to improve the process [3]. Therefore, achieving long-term expansion and dry maintenance of dental MSCs is crucial for ensuring the number, quality, and efficacy of stem cells [16].

Significant progress has been made in understanding stem cells, the genes that control their fate, and the niches that provide signals to modulate their decisions [2, 3]. While several studies have been conducted on the role of dental MSCs in regenerative dentistry, relatively few have summarized the effects of different GFs on the application of human dental MSCs. This review aims to systematically examine research on multi-mode treatment of human dental MSCs with GFs in the past decade to determine the effect of single GFs and their specific receptors or mechanisms. It also investigates the effects of multiple GFs combined with dental MSCs and specific receptors or mechanisms. This paper can provide valuable insights into the development of human dental MSCs regenerative medicine and clinical application.

## Methods

This systematic review followed the Preferred Reporting Items for Systematic Reviews and Meta-Analyses (PRISMA 2020) guidelines.

### Search strategy

The systematic review utilized The databases Web of Science core collection and PubMed databases due to their high quality and abundance of relevant articles. The reviewers searched the Web of Science core collection database using the following terms: “dental mesenchymal stem cell*” OR “periodontal ligament stem cell*” OR “dental follicle stem cell*” OR “stem cell from human exfoliated deciduous teeth” OR “dental pulp stem cell*” OR “stem cells of the apical papilla” OR “gingival mesenchymal stem cell*” AND “Receptors, Growth Factor”. At the same time, the PubMed database was used to perform “Majr” of “Receptors, Growth Factor”. Nearly 10 years of English works of literature from 2014 to 2023 were screened. Moreover, the final selection was made on the 15th of July 2023.

### Eligibility, inclusion, and exclusion criteria

The study included experimental articles that provided information on the effects of GFs during the application of human dental MSCs. The following literature was excluded: nonhuman, no open access, reviews, retracted, unspecified, meeting, and other topics (studies that do not treat with GFs, genetic, plasmidor adenoviruses treated, impact factors < 2.5). After screening the full text of the eligible articles, only papers focusing on the effects of GFs during the application of human dental MSCs were included. The reviewers independently studied the screening records and read the full text of each article to identify potentially qualified and relevant studies. This method allowed for the analysis of the content of a manuscript that meets the requirements. Only the literature that fulfilled the inclusion criteria was selected for this review.

### Data extraction

The reviewers independently collected outcomes related to two aspects - single GF and multiple GFs. Firstly, the assessment of publication year and types of dental MSCs was completed. The unique parameters and information about the authors’ names, studies, GFs, dental MSCs, receptors, mechanisms, and effects were further extracted to evaluate the efficacy outcomes.

## Results

### Design and samples

**Fig. 1:**
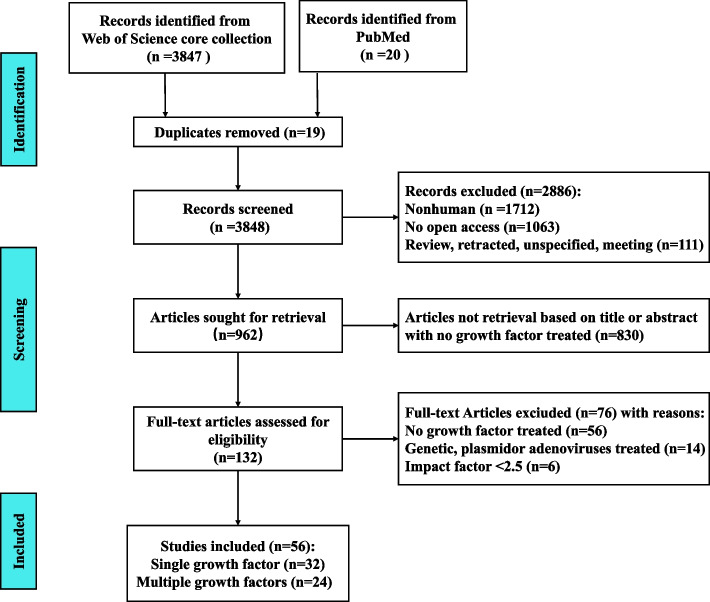
Flow chart of the literature search

In this systematic review, 3847 records were initially obtained through database searching from the Web of Science core collection, and 20 records from PubMed were included. After excluding 2886 manuscripts due to duplication, non-human content, lack of open access, reviews, untraceable sources, unspecified content, and meetings, only 19 records remained for screening. After reviewing the titles and abstracts of 962 articles, 830 were excluded because they did not cover GFs. The full text of the remaining 132 articles was analyzed, and 56 were selected for their focus on the effects of GFs for treating human dental MSCs. Out of those 56 articles, 32 focused on a single growth factor while 24 focused on multiple growth factors. A flow chart illustrating this process is presented in Fig. 1.

### The study characteristics

**Fig. 2:**
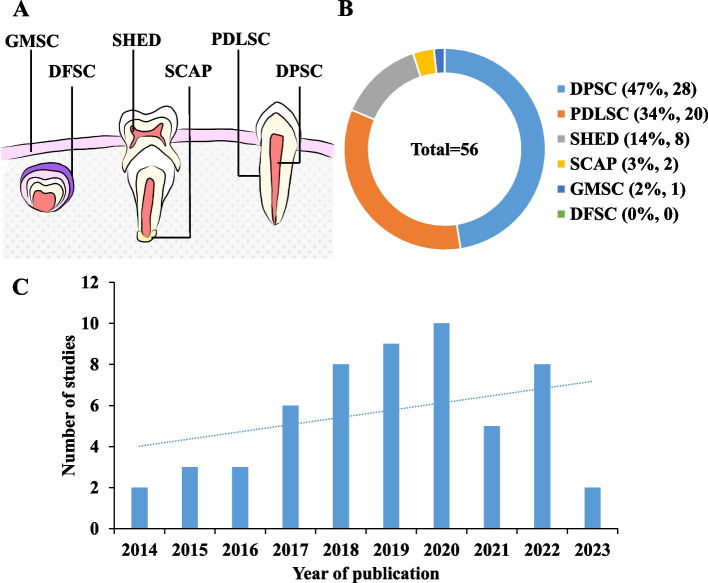
Study characteristics. **A** human dental MSCs types. (DPSC: dental pulp stem cell, PDLSC: periodontal ligament stem cell, SHED: stem cell from human exfoliated deciduous teeth, SCAP: stem cells of the apical papilla, GMSC: gingival mesenchymal stem cell, DFSC: dental follicle stem cell) **B** Proportion of human dental MSCs types treated by GFs in the selected studies. **C** Publication year of the selected studies on human dental MSCs treated by GFs

For this systematic review, a total of 56 studies were selected. Human dental MSCs have garnered increasing attention from scholars due to their unique advantages in the field of regenerative medicine. This has made it a focal point for future research. According to the review, 47% of the articles were about DPSC, while 34% covered PDLSC, SHED, SCAP, and GMSC to a lesser extent. However, there were no studies on DFSC. This indicates that DPSC and PDLSC have the most promising applications in human dental MSCs. Over the past decade, research on GFs treatment of human dental MSCs has shown a gradual increase, with only two papers meeting the subject requirement in 2014, but increasing to ten in 2020 (Fig. 2).

### The application of a single GF

This review covers 32 literary works using human dental MSCs with single GF treatment. The research mainly examines the therapeutic effects of different GFs on dental MSCs. However, there are only a few studies on the corresponding body or specific mechanism. The literature commonly uses pathway inhibitors to validate receptors and mechanisms. Table 1 presents detailed information about growth factors (GFs), cell types, receptors, pathways, and effects.

**Table 1:** The therapeutic effect of human dental MSCs treated by single GF

Literature	GFs	Cell types	Receptors	Pathways	Effects
Chun Fan et al. [17]	TGF-β1	PDLSC	-	ROS	induce aging
Hsiao-Hua Chang et al. [18]	TGF-β1	SHED	TGF-βRI, TGF-βRII	ALK5/Smad2, TAK1, p38, MEK/ERK	promote proliferation, collagen turnover, and differentiation
Liming Jiang et al. [19]	TGF-β1	DPSC	-	-	promote pulp regeneration or restorative dentin formation
Parisa Ghandforoushan et al. [20]	TGF-β1	DPSC	-	-	promote adhesion, proliferation, and differentiation of chondrocytes
Alireza Moshaverinia et al. [21]	TGF-β3	PDLSC, GMSC	-	-	promote tendon repair and regeneration
Yangfan Li et al. [22]	TGF-β3	PDLSC	-	-	promote osteogenic differentiation and repair incomplete bone defects
Jingting Lu et al. [23]	FGF9	DPSC	-	ERK1/2	inhibit osteogenic differentiation
Caroline Gorin et al. [24]	FGF-2	SHED	-	-	induce the release of VEGF and HGF and enhance the angiogenesis potential
Anita Novais et al. [25]	FGF-2	SHED	-	-	increase the bone healing potential
Chunshu Zhang et al. [26]	FGF-2	PDLSC	-	-	promote proliferation, dry expression, and cytokine secretion
Jessica Ratajczak et al. [27]	FGF-2	PDLSC	-	-	promote angiogenesis secretion
J Qian et al. [28]	bFGF	DPSC	-	-	treatment for 1 week to increase bone formation, treatment for 2 weeks to reduce bone formation
Lihua Luo et al. [29]	bFGF	DPSC	-	ERK, TRPC1	save the proliferative activity of frozen cells without changing the dry and pluripotency
Nunthawan Nowwarote et al. [30]	bFGF	SHED	-	Pi/PPi metabolism	increase the number of cells and maintain stem cell characteristics
Casiano Del Angel-Mosqueda et al. [31]	EGF	DPSC	-	-	promote extracellular matrix mineralization, osteogenic differentiation
	bFGF	DPSC	-	-	inhibit osteogenic differentiation
De-Hua Zheng et al. [32]	EPO	PDLSC	-	Wnt/β-catenin	dose-dependent contributes to bone differentiation
Liying Wang et al. [33]	EPO	PDLSC	-	p38 MAPK	promote proliferation and osteogenic differentiation
Ji Hoon Park et al. [34]	BMP peptide	DPSC	-	-	support high cell viability, accelerates proliferation and odontogenic differentiation
Selen Küçükkaya Eren et al. [35]	BMP-7	DPSC	-	-	increase osteogenic differentiation and regeneration
Cheng Liang et al. [36]	BMP7	DPSC	-	-	promote vascular regeneration in a concentration-dependent manner
	TGF-β1	DPSC	-	-	completely inhibits calcification,
Seung Hun Park et al. [37]	BMP2	PDLSC	-	-	promote osteogenic differentiation non-invasively
Edit Hrubi et al. [38]	BMP2	DPSC	BMPRI, BMPRII	-	inhibit cell proliferation, and use alone is not sufficient to induce osteogenesis
Joo-Young Park et al. [39]	BMP-2	PDLSC	-	-	higher mineralization and collagen synthesis
Qian Zeng et al. [40]	CGF	DPSC	-	-	promote pulp healing
Joshua N Winderlich et al. [41]	VEGF-a	DPSC	VEGF-R2	-	increase the permeability of the blood–brain barrier, stimulate the adhesion and migration of cells
J G Xu et al. [42]	VEGF-a	SHED, DPSC	-	SMAD2/3	enhance endothelial differentiation
Nan Xiao et al. [43]	GDNF	DPSC	GFR	AKT, MAPK	increase migration and promote rapid wound healing
Arwa A Al-Maswary et al. [44]	BDNF	DPSC	-	ERK/MAPK	promote differentiation into typical neuron-like cells
Saikrishna Kandalam et al. [45]	BDNF	SCAP[55]	-	-	induce immune regulation, protect nerves, and promote the expression of neuronal markers
Ji-Hyun Kim et al. [46]	BDNF	DPSC	TrkB	-	induce odontogenic differentiation
Zhenqing Liu et al. [47]	NGF	-	p75NTR	JNK	activation of the DLX5 gene contributes to bone

-unknown or not mentioned

*DPSC* dental pulp stem cell, *PDLSC* periodontal ligament stem cell, *SHED* stem cell from human exfoliated deciduous teeth, *SCAP* stem cells of the apical papilla, *GMSC* gingival mesenchymal stem cell, *TGF-β* transforming growth factor β, *FGF* fibroblast growth factor, *bFGF* basic fibroblast growth factor, *EGF* epidermal growth factor, *EPO* erythropoietin, *BMP* bone morphogenetic protein, *VEGF* vascular endothelial growth factor, *GDNF* glial-derived neurotrophic factor, *BDNF* brain derived growth factor, *NGF* nerve growth factor, *CGF* Concentrated growth factor, *ROS* reactive oxygen species

### The application of multiple GFs

In this review, 24 pieces of literature focus on the effects of different GFs on human dental MSCs. The combination of multiple GFs is more complex when compared to the treatment of just one GF. The previous studies mainly focused on the efficacy of dental MSCs combined with GFs and rarely investigated the corresponding receptors or pathways. Table 2 provides detailed information on GFs, cell types, receptors, pathways, and their effects.

**Table 2:** The therapeutic effect of human dental MSCs treated by multiple GFs

Literature	GFs	Cell types	Receptors	Pathways	Effects
Sun-Yi Hyun et al. [48]	FGF-2, TGF-β1, BMP-2/-4	PDLSC	-	-	FGF-2 collaborates with TGF-β1 to stimulate fibrotic differentiation and antagonize BMP osteogenic/cemental differentiation
Nan Xiao et al. [49]	BDNF, NT4/5	DPSC	TrkB	ERK/MAPK	accelerate migration and wound healing
Wanyu Lu et al. [50]	IGF-1,VEGF	DPSC	-	AKT	combined to promote proliferative migration and osteogenesis, the effect alone is not obvious
Kun Xia et al. [51]	RGD, VEGF	DPSC	-	-	promote cell adhesion, angiogenesis, and endodontic regeneration
Francesco Paduano et al. [52]	Medium (EGF, bFGF)	DPSC	-	-	up-regulate osteogenesis-specific markers
Anna Di Vito et al. [53]	Medium (EGF, FGF)	PDLSC	-	-	maintain growth and dryness with higher osteogenic potential
Jingyi Xiao et al. [54]	Medium (FGF2, TGF β1)	DPSC	-	-	higher maintenance of cell proliferation, pluripotency, migration, and stability
Jialin Chen et al. [55]	Medium (bFGF-2, TGF-β3, SP)	PDLSC	-	-	construction of multilayer human corneal stromal-like tissue
Wendy Martens et al. [56]	Medium (PDGF-aa, bFGF, NRG)	DPSC	-	-	induce differentiation into Schwann-like cells
A Longoni et al. [57]	Medium (TGF-β3, BMP-2/-6/-7, IGF-1)	DPSC	-	-	fibrocartilaginous tissue is formed, hyaline cartilage is not formed
Huong Thi Nguyen Nguyen et al. [58]	Medium (EGF, bFGF, BDNF)	SHED	-	-	induction into neurons improves neurite development and mitochondrial function
Xu, JG et al. [59]	Medium (TGF-β1, BMP4)	SHED	-	TGF-β1-ALK5	derived to SMC
Hua-Lian Cao et al. [60]	AFC	DPSC, PDLSC	-	-	GF source that promotes dentin/dentin differentiation, cell expansion
Prakan Thanasrisuebwong et al. [61]	i-PRF	PDLSC	-	-	yellow i-PRF stimulates osteogenic differentiation earlier, and red i-PRF is more suitable for bone regeneration
Melissa Lo Monaco et al. [62]	L-PRF	DPSC	-	-	an immunomodulatory effect, stimulate the survival of chondrocytes
Ali Sadeghinia et al. [63]	a-PRP	DPSC	-	-	accelerate cell osteogenic differentiation, mineralization, and expression of bone gene markers
Yunhe Xu et al. [64]	PRP	PDLSC	-	autophagy	concentration-dependent enhancement of cell viability and osteogenic differentiation
Qiu Xu et al. [65]	PRP	PDLSC	-	-	significantly enhances osteogenesis, with a concentration of 1% being the most effective mode of administration
Bei-Min Tian et al. [66]	PL	PDLSC	-	-	improve the osteogenic potential and support cell sheet formation
Gengtao Qiu et al. [67]	PL	PDLSC			enhance osteogenic differentiation potential
Gengtao Qiu et al. [68]	PL	PDLSC	-	-	improve cell viability and osteogenic differentiation, 2.5% is the optimal concentration
Nela Pilbauerova et al. [69]	PL	DPSC	-	-	serum substitute for expanded stem cells in vitro
Hanan Jafar et al. [70]	PL	SCAP, PDLSC	-	-	a suitable substitute for animal-derived serums that contribute to bone
Tong Lei et al. [71]	PL	SHED	-	-	promote stem cell proliferation and differentiation, and standardize cell production methods

-unknown or not mentioned

*DPSC* dental pulp stem cell, *PDLSC* periodontal ligament stem cell, *SHED* stem cell from human exfoliated deciduous teeth, *SCAP* stem cells of the apical papilla, *SMC* smooth muscle cell, *TGF-β* transforming growth factor β, *FGF* fibroblast growth factor, *bFGF* basic fibroblast growth factor, *EGF* epidermal growth factor, *BMP* bone morphogenetic protein, *SP* substance P, *VEGF* vascular endothelial growth factor, *BDNF* brain derived growth factor, *NT* neurotrophin, *IGF* insulin-like growth factor, *AFC* Allogeneic Fibrin Clot, *PRF* platelet-rich fibrin, *PRP* platelet-rich plasmawithin, *PL* platelet lysate

### Mechanism of GFs acting on human dental MSCs

**Fig. 3:**
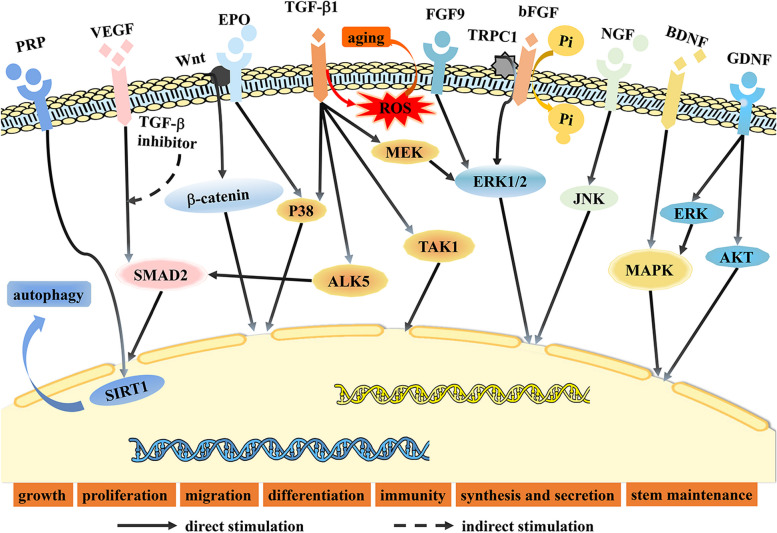
Mechanism of GFs acting on dental MSCs. (PRP: platelet-rich plasmawithin, VEGF: vascular endothelial growth factor, EPO: erythropoietin, TGF-β: transforming growth factor β, ROS: reactive oxygen species, FGF: fibroblast growth factor, bFGF: basic fibroblast growth factor, NGF: nerve growth factor, BDNF: brain derived growth factor, GDNF: glial-derived neurotrophic factor)

How GFs work is not straightforward. Although different GFs can affect dental MSCs using the same signaling pathway, a single GF can also use several signaling pathways to influence dental MSCs. As per the data gathered so far, various GFs affecting specific receptors or signaling pathways on dental MSCs are illustrated in Fig. 3.

## Discussion

This review aims to systematically analyze how GFs regulate human dental MSCs through multi-way processing. Dental MSCs have become increasingly significant as they possess therapeutic abilities in treating various diseases without causing any serious adverse effects [72]. According to A.A. Volponi et al. [73], dental MSCs from various sources possess unique functional characteristics, which is in line with the findings of Dean Whiting et al. [74].

### A single GF

Different GFs have varying effects on dental MSCs. FGF-2 treatment increases the proportion of Stro-1 + /CD146 + progenitor cells in SHED and improves vascularization differentiation efficiency more than hypoxia [24]. Similarly, treating DPSC with BDNF using the traditional SH-SY5Y sequential method can lead to more noticeable neuron-like characteristics [44]. These results suggest that supplementing with GFs can be a potential therapy for regenerating human dental MSCs in the pulp, blood vessels, and nerves. The effects of GFs may vary depending on the concentration used. J Qian et al. [28] discovered that the ability of DPSC to undergo osteogenic differentiation increased after 1 week of bFGF pretreatment. Although the effectiveness of bFGF is not diminished by passage, the osteogenic impact is reduced after 2 weeks of preconditioning, which is consistent with the findings of Casiano Del Angel-Mosqueda et al. [31].

A single GF can have multiple biological roles. The effect of bFGF on DPSC is not limited to osteogenesis but also helps to up-regulate the TRPC1 channel, which prevents apoptosis. Continuous treatment of Stem Cells from SHED with bFGF plays a vital role in regulating Pi/PPi metabolism and maintaining stem cell properties [28–30]. GFs can also have the same impact on dental MSCs. For instance, NGF and EGF both have osteogenic effects, and BMP2 and FGF9 are also capable of inhibiting osteogenic differentiation. This could provide the basis for the combined application of GFs [23, 31, 38, 47].

### Multiple GFs

When comparing single GF treatment to combined GF treatment, it can have either a synergistic or antagonistic effect. The combination of IGF-1 and VEGF works through the AKT pathway to stimulate DPSC growth. FGF-2 can work with TGF-β1 to stimulate PDLSC differentiation but antagonize BMP-induced differentiation [48, 50]. The use of multiple GFs has proven to be a better alternative to traditional stem cell culture medium. EGF, bFGF, and BDNF in a medium can stimulate dopaminergic neuron formation [58, 59]. DPSC can induce Schwann-like cells using PDGF-aa, bFGF, NRG, TGF-β3, BMP-2/-6/-7, and IGF-1 [56, 57]. PDLSC, on the other hand, can differentiate into corneal cells using bFGF-2, TGF-β3, and SP. Lastly, an EHFM medium is the best option for the long-term expansion of PDLSC [53, 55].

Different human platelet derivatives contain natural GFs with various effects. PRP enhances cell viability in PDLSC while PL has higher activity of GFs and fewer side effects [64, 65, 68–70]. AFC is also a safe alternative to serum with a high content of GFs [60]. In i-PRF, yellow i-PRF stimulates osteogenic differentiation earlier, but red i-PRF is more suitable for bone regeneration [61]. However, GFs in vivo have limitations, improving their delivery systems can extend their stability and lifespan [75]. Hydrogel materials and modified gels enable tissue engineering with GFs like FGF, BMP, VEGF, and TGF-β [21, 25, 51, 66, 71]. Mineral trioxide aggregate (MTA), root BP Plus, and doxycycline (DOX) play an auxiliary role in the interaction process [35, 40, 67].

### Receptors

The mechanism by which GFs act on human dental MSCs is complex but critical. The receptors related to the TGF-β1 signal, such as TGF-βRIs (ALK1, ALK3, ALK5), TGF-βRII, β glycan, and endothelial glycoproteins, are detectable in SHED. Type I and II receptors within SHED enhance collagen synthesis, and TGF-β1 further affects SHED through differentially regulating ALK5/Smad2/3, TAK1, p38, and MEK/ERK pathways [18]. At a molecular level, TGF-β1 promotes the activity of β-galactosidase and the expression of p16 and p21 in PDLSC, leading to cellular senescence due to excessive ROS generation [17]. As for DPSC, researchers believe that BMP2, VEGF-a, GDNF, and BDNF mediate cell proliferation, migration, and differentiation through specific receptors [38, 41, 43, 46]. These findings suggest that GFs initiate biological processes by activating particular receptors in dental MSCs.

### Limitations

It is important to acknowledge certain limitations in this study. The search strategy only considered literature from the last decade, which means that not all available literature was included. Additionally, the included research mainly focused on basic research of cells or the primary animal model used in mice, which increases the risk of bias and confounding. This article mainly discusses dental MSCs, types of GFs, and specific uses in regenerative medicine, while ignoring the following aspects: (1) age, sex, and viability of human dental MSC donors; (2) number of animal experiments and duration of intervention, and (3) statistical methods used. Dental MSCs show great promise in regenerative medicine. However, the literature does not clarify the mechanism of interaction between different GFs in detail. Potential biases in the data may have further affected the systematic analysis. Due to the lack of a clinical database, a meta-analysis was not performed.

Therefore, to strengthen the conclusion, future improvements can be made in the following areas. First, conducting additional research that includes experiments and data from a broader range of species such as pigs, dogs, or humans is necessary. Second, it is important to consider the potential for publication, ensuring an adequate description of the mechanisms governing interaction between different growth factors in human dental MSCs. More consistent use of statistical methods, age, sex, and viability of human dental MSC donors, along with inhibition of experimental intervention can lead to higher-quality articles.

## Conclusion

Multimodal treatment of GFs can effectively regulate human dental MSCs, ensuring stem cell quality, quantity, and curative effects.

### Supplementary Information

**Additional file 1. **PRISMA 2020 Checklist.

## Data Availability

The datasets supporting the conclusions of this article are included within the article and its Additional file 1.

## Abbreviations

MSCs: Mesenchymal stem cells
GF: Growth factor
PDLSC: Periodontal ligament stem cell
DFSC: Dental follicle stem cell
SHED: Stem cell from human exfoliated deciduous teeth
DPSC: Dental pulp stem cell
SCAP: Stem cells of the apical papilla
GMSC: Gingival mesenchymal stem cell
EGF: Epidermal growth factor
TGF-β: Transforming growth factor β
PDGF: Platelet-derived growth factor
bFGF: Basic fibroblast growth factor
BMP: Bone morphogenetic protein
IGF: Insulin-like growth factor
VEGF: Vascular endothelial growth factor
NGF: Nerve growth factor
EPO: Erythropoietin
GDNF: Glial-derived neurotrophic factor
BDNF: Brain derived growth factor
NT: Neurotrophin
SP: Substance P
PDGF: Platelet-derived growth factor
CGF: Concentrated growth factor
SMC: Smooth muscle cells
BBB: Blood–brain barrier
PL: Platelet lysate
AFC: Allogeneic Fibrin Clot
PRF: Platelet-Rich Fibrin
PRP: Platelet-rich plasmawithin
ROS: Reactive oxygen species
3D: Three-dimensional
DOX: Doxycycline
MTA: Mineral trioxide aggregate
